# Ongoing increase in autochthonous *Salmonella* Enteritidis infections, the Netherlands, June 2023 to June 2025

**DOI:** 10.2807/1560-7917.ES.2025.30.30.2500536

**Published:** 2025-07-31

**Authors:** Oda E van den Berg, Dana LL Adriaansens, Maren I Lanzl, Kirsten Mooijman, Angela HAM van Hoek, Diederik AH Brandwagt, Sabiena G Feenstra-Gols, Joline Mans-Poulie, Petra Y Dop, Ife A Slegers-Fitz-James, Maaike van den Beld, Roan Pijnacker, Eelco Franz

**Affiliations:** 1 Centre for Infectious Disease Control, National Institute for Public Health and the Environment (RIVM), Bilthoven, The Netherlands; 2 Netherlands Food and Consumer Product Safety Authority (NVWA), Utrecht, The Netherlands

**Keywords:** Salmonella *Enteritidis*, poultry, poultry products, outbreak, whole genome sequencing, The Netherlands

## Abstract

*Salmonella* Enteritidis (SE) cases in humans have increased in the Netherlands, from an annual average of 281 (2017–2019) to 427 in 2023, 401 in 2024 and 209 in 2025 (January–June). This rise is paralleled by a 2.5-fold increase in SE-positive laying hen flocks. Genomic surveillance shows numerous small clusters, suggesting a diffuse transmission rather than a single point-source outbreak, which complicates outbreak investigations. Coordinated interventions in the laying hen sector are urgently needed to mitigate public health risks.

Since mid-2023, the Netherlands has experienced a strong and sustained increase in human *Salmonella enterica* serovar Enteritidis (SE) infections. This rise coincides with an increase in SE-positive laying hen flocks and presents a growing public health concern. Here, we describe the recent genomic and epidemiological patterns of SE in humans and the laying hen sector in the Netherlands and discuss current challenges in response.

## Laboratory-confirmed infections in humans

Surveillance of salmonellosis in humans is performed through the Dutch national laboratory surveillance network at the National Institute for Public Health and the Environment (RIVM). Medical microbiology laboratories voluntarily send *Salmonella* isolates to the RIVM for further typing using whole genome sequencing (WGS).

**Figure 1:**
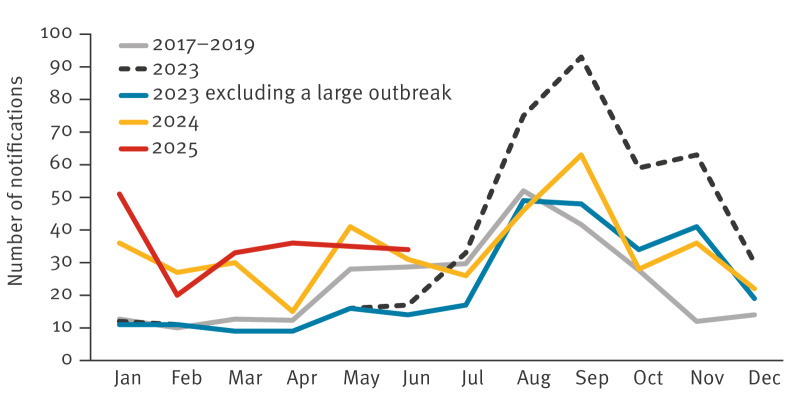
Number of laboratory-confirmed cases of *Salmonella* Enteritidis in humans, by month of isolate receipt at RIVM, the Netherlands, 2017–June 2025 (n = 1,881)^a^

**Figure 2:**
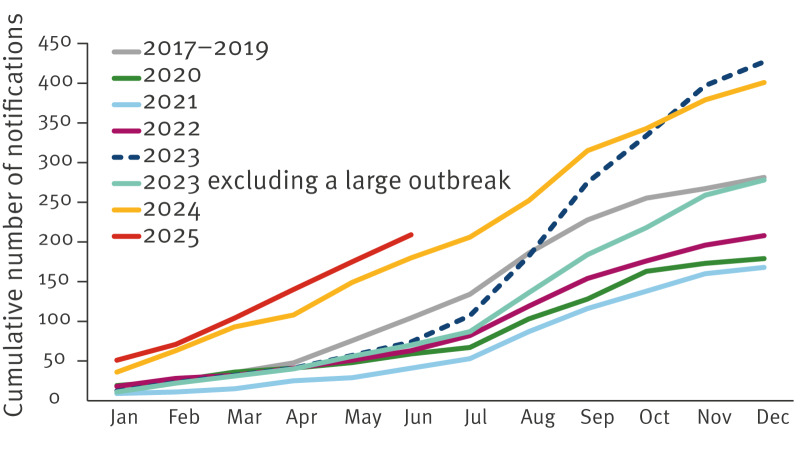
Cumulative number of laboratory-confirmed cases of *Salmonella* Enteritidis in humans, by month of isolate receipt at RIVM, the Netherlands, 2017–June 2025 (n = 2,436)^a^

Here, we present case numbers excluding those associated with foreign travel. Travel-associated cases include cases with reported recent travel, cases from clusters of at least 10 patients in which > 20% had a travel history (noting that travel history is often missing), and cases originating from specific foreign laboratories. In 2023, the number of laboratory-confirmed SE cases rose to 427 (population of the Netherlands: 18 million people) from an annual average of 281 cases (range: 246–333) between 2017 and 2019, i.e. pre-COVID (Figure 1, Figure 2). In 2024, a total of 401 cases were notified. We observed a similar pattern in the first 6 months of 2025, when 209 cases were notified, compared with 180 during the same period in 2024, and an annual average of 104 cases (range: 94–119) during the first 6 months of 2017–2019.

RIVM: the National Institute for Public Health and the Environment, the Netherlands.

Source: RIVM.

^a^ Cases considered to be associated with foreign travel and cases from pandemic years 2020–2022 were excluded.

The grey line depicts the average number of cases 2017–2019. Additionally, in the solid line of 2023, patients related to a large *Salmonella* Enteritidis outbreak have been excluded.

RIVM: the National Institute for Public Health and the Environment, the Netherlands.

Source: RIVM.

^a^ Cases considered to be associated with foreign travel were excluded.

The grey line depicts the average number of cases 2017–2019. Additionally, in the solid line of 2023, patients related to a large *Salmonella* Enteritidis outbreak have been excluded.

The increase in 2023 was largely due to a major outbreak, likely linked to eggshells from SE-positive layer flocks, inadequately treated before being ground and added to poultry feed. This likely led to a widespread infection in the laying hen sector and subsequently resulted in human cases through the consumption of contaminated eggs [1,2]. This outbreak, which began in the first half of 2023, has resulted in 151 cases in 2023, 27 in 2024 and 31 in 2025 (until June 2025).

The true number of cases is likely much higher than the number reported, as generally more patients are affected than we receive isolates of, with an estimated overall under-reporting factor of 26 [3].

## Whole genome sequencing of isolates from humans

Clusters were identified by WGS analysis using core genome multilocus sequence typing (cgMLST) with 3,002 targets (Enterobase scheme; https://enterobase.warwick.ac.uk). Clusters were defined using a 5-allele cutoff under a single-linkage clustering algorithm.

Following the major outbreak in 2023, most cases in 2024 and 2025 have occurred in smaller clusters. In the WGS cluster analysis, we identified 38 non-travel-related SE clusters in 2023, 42 in 2024 and 22 in 2025 (Jan-Jun), with a median size of three cases for all years (ranges: 2–148 in 2023, 2–45 in 2024 and 2–35 in 2025). The Simpson diversity index rose from 0.79 in 2023 to 0.94 in 2024 and to 0.87 in 2025 (until June), indicating increased diversity and more small clusters in 2024–2025. This complicates outbreak investigations and hinders the identification and control of infection pathways.

An alert was issued in EpiPulse (an online portal for European public health authorities and partner organisations and maintained by the European Centre for Disease Prevention and Control (ECDC)) (item number: 2024-FWD-0021) about the increase in SE in the Netherlands on 5 April 2024. To date, this rise in human cases appears to be country-specific, as no other European Union/European Economic Area (EU/EEA) country has reported a similar increase.

## *Salmonella* Enteritidis in laying hen flocks

**Figure 3:**
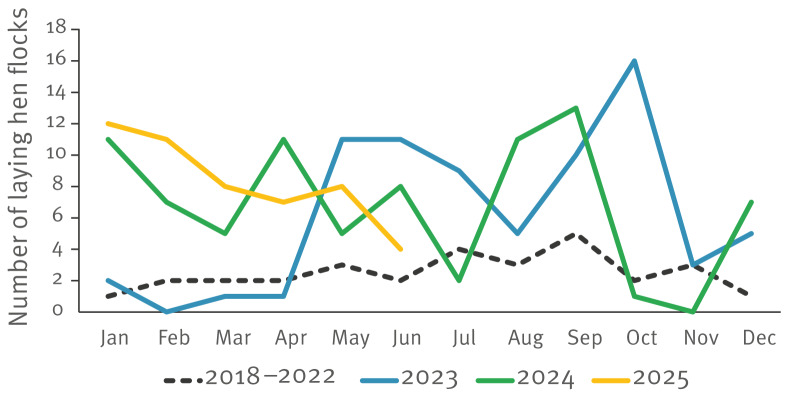
Number of laying hen flocks positive for *Salmonella* Enteritidis, by month, the Netherlands, 2018–June 2025 (n = 355)

NVWA: Netherlands Food and Consumer Product Safety Authority; SE: *Salmonella* Enteritidis.

Source: NVWA.

Information on *Salmonella* in laying hen flocks was collected through the national Animal Health Monitoring programmes of the Netherlands Food and Consumer Product Safety Authority (NVWA). In accordance with the EU regulations [4], adult laying flocks are sampled at least every 15 weeks during the laying period, starting from 24 ± 2 weeks of age. This monitoring showed an increase in SE-positive laying hen flocks since May 2023, paralleling the increase in humans (Figure 3). In 2023, 74 SE-positive flocks were detected, followed by 81 in 2024 and 50 in 2025 (Jan-Jun). Between 2018 and 2022, an average of 30 positive flocks were detected annually. While most pronounced in the Netherlands, the European Food Safety Authority (EFSA) data up to 2023 also show increases in SE-positive laying hen flocks in several other European countries, although with a lower prevalence than in the Netherlands [5].

The dotted line depicts the average number of laying hen flocks positive for SE 2018–2022.

## Time-lagged association between *Salmonella *Enteritidis in humans and laying hen flocks

To further investigate the relationship between SE case numbers in humans and SE-positive laying hen flocks, we performed time-series analyses. We observed a significant correlation between the monthly number of SE-positive laying hen flocks and notified SE cases in humans (Pearson’s r = 0.46; p = 0.005). Granger causality analysis indicated that the number of SE-positive laying hen flocks significantly predicted the number of human SE cases at time lags of 1 month (F = 4.3; p = 0.046), as well as at lags of 3–6 months (p = 0.048; p = 0.032; p = 0.024; p = 0.030). This underscores a time-dependent association between SE infections in the laying hen sector and the surge in human SE cases.

## Discussion

The implementation of EU-harmonised *Salmonella* control programmes in poultry, the primary source of human SE infection, has led to a marked decline in SE incidence in humans across Europe since the early 2000s, mainly due to regulations requiring eggs from SE-positive flocks to be diverted for industrial use. On Dutch laying hen farms, culling is not mandatory after detection of SE, but all eggs from positive flocks must go for processing including a heat treatment. After the production cycle, the house must be thoroughly cleaned and disinfected before new hens can be placed. Nevertheless, delays between routine flock testing and SE detection can occasionally result in contaminated eggs being sold as table eggs. By 2013–2014, the decrease in incidence rates stabilised across the EU and incidence rates even increased in the Netherlands since 2015 [6-8]. Since mid-2023, the Netherlands has experienced a stronger and sustained increase in human SE infections.

Due to the ongoing, diffuse increase in SE cases in humans and laying hen flocks, the RIVM organised a Response Team Zoonoses (RT-Z) in early 2025 via the established zoonoses structure [9]. The aim of this RT-Z was to characterise and interpret the SE rise in an integrated One Health approach to assess the public health risks and to advice on the strategy to control spreading. The presence of numerous small clusters suggests a diffuse transmission pattern rather than a single point-source outbreak. This limits the usefulness of traditional outbreak investigations, which tend to identify only individual farms. As a result, the recommended control measures shifted from targeting single farms to taking a broader, sector-wide approach focusing on reducing SE prevalence and transmission throughout the industry. The RT-Z advised several possible interventions, which include (i) increase testing frequency in laying hen flocks to reduce the period in which contaminated eggs are inadvertently marketed, helping to limit human exposure; (ii) sequence more SE isolates from holdings with positive flocks of laying hens, followed by cross-sectoral analysis to understand transmission pathways (including environment or feed); (iii) accelerate removal of SE-positive flocks to lower infection pressure in the laying sector as a whole, reducing risk of further farm-to-farm spread and re-infection.

The reasons for the sustained increase in SE prevalence in the laying hen sector remain unclear. One possibility is the extended productive lifespan of laying hens, which may increase their vulnerability to SE as vaccine-induced immunity wanes over time. In addition, the higher prevalence and prolonged SE positivity in flocks may increase infection pressure within and between farms, creating a lasting reservoir for (re)infection and facilitating farm-to-farm transmission. The outbreak that began in June 2023 was most likely triggered by contaminated eggshells from SE-positive farms used as a calcium source in poultry feed [1, 2]. Although heat treatment is standard practice to eliminate *Salmonella* from eggshells before their use in feed*,* investigations revealed that some eggshells were inadequately treated, possibly allowing *Salmonella *to spread widely and act as a super-spreading event of SE in laying hen flocks in the Netherlands.

## Conclusion

The ongoing rise in SE cases in humans and laying hen flocks in the Netherlands presents a significant public health risk. The widespread, diffuse transmission calls for coordinated, cross-sectoral control strategies and more integrated surveillance. While the increase currently appears limited to the Netherlands, spread to other European countries is possible due to trade, travel and movement of poultry products. Coordinated action is essential to unravel underlying causes of the SE increase in laying hen holdings in the Netherlands and to direct effective interventions to reduce the number of human SE cases.

## Data Availability

The data that support the findings of this study are available from the corresponding author upon reasonable request.
